# Less Is More? Physical Inactivity and Increased Risk of Diabetes‐Related Foot Ulceration—A Systematic Review

**DOI:** 10.1002/dmrr.70198

**Published:** 2026-07-06

**Authors:** Petra J. Jones, David G. Armstrong, Alex V. Rowlands, Harald Sourij, Afsaneh Noormandi, George Theodorakopoulos

**Affiliations:** ^1^ Leicester Diabetes Centre Leicester General Hospital University Hospitals of Leicester Leicester UK; ^2^ Diabetes Research Centre Leicester General Hospital University of Leicester Leicester UK; ^3^ NIHR Leicester Biomedical Research Centre Leicester General Hospital Leicester UK; ^4^ Keck School of Medicine University of Southern California Los Angeles California USA; ^5^ Alliance for Research in Exercise, Nutrition and Activity (ARENA) Adelaide University Adelaide Australia; ^6^ Cardiometabolic Trials Unit, Division of Endocrinology and Diabetology Medical University of Graz Graz Austria; ^7^ Technological Educational Institute of Patras Patras Greece; ^8^ Queen Margaret University Edinburgh UK; ^9^ University of West Attica Aigaleo Greece; ^10^ University of Thessaly Larisa Greece; ^11^ National and Kapodistrian University of Athens Athens Greece; ^12^ Center to Stream Healthcare in Place (C2SHiP) Alexandria Virginia USA

**Keywords:** diabetes, foot, inactivity, physical activity, sedentary, ulcer

## Abstract

**Aims:**

Those at risk of diabetes‐related foot ulceration (DFU) must balance sufficient physical activity to maintain glycaemic control and cardiovascular health whilst avoiding excessive trauma to their feet. Our systematic review aimed to assess whether physical inactivity and/or sedentary behaviour affect DFU outcomes.

**Materials and Methods:**

Embase, Medline, and Scopus were searched for peer‐reviewed studies using the criteria: (‘diabetes’ OR ‘diabetic’) AND (‘physical*’ OR ‘activ*’ OR ‘inactiv*’ OR ‘sedentary’) AND ‘foot’ AND ‘ulcer’, returning 4650 results excluding duplicates. 16 studies were included and assessed for risk of bias using the Newcastle‐Ottawa Scale (NOS).

**Results:**

Fifteen studies assessed physical inactivity and 1 assessed both inactivity and sedentary behaviour and DFU outcomes. Eleven of 16 (69%) studies reported significantly greater likelihood of DFU in inactive participants. Exploratory meta‐analysis suggested physical inactivity to be associated with doubled DFU risk (OR 2.09, 95% CI 1.32–3.32, *p* = 0.002). Evidence that sedentary behaviour was associated with tripled DFU risk was based on a single prospective cohort and had a high risk of bias (OR 2.95, 95% CI: 1.5–6.4, *p* = 0.008; *n* = 175, NOS: poor). Study methodologies were heterogenous (e.g., inconsistent definitions of inactive), with habitual physical activity measured through interviews, patient records, or questionnaires in 14 of 16 studies (NOS rating: poor to fair).

**Conclusions:**

Available evidence suggested that physical inactivity and sedentary behaviour were associated with an increased risk of DFU. Further research is needed to develop thresholds for physical inactivity and sedentary behaviour to reduce DFU risk.

## 1 Introduction

The number of people living with diabetes worldwide was already 589 million in 2025 [1] and is growing. Foot ulceration is a serious complication of diabetes that can double both the risk of amputation [2] and 5‐year mortality [3] with a substantial adverse effect on quality of life [4]. The global economic healthcare burden of diabetes‐related foot ulceration (DFU) is considerable [5, 6], yet a surprising number of fundamental questions remain about physical activity and the risk of developing diabetes‐related foot ulcers.

**FIGURE 1:**
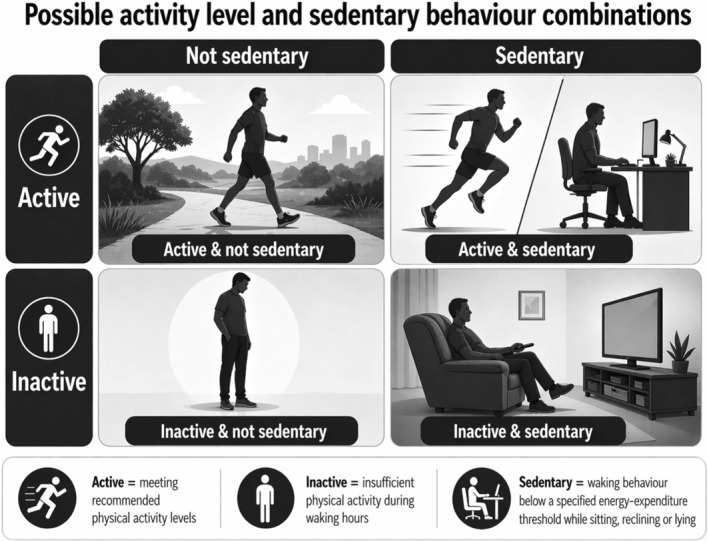
Possible activity level and sedentary behaviour status combinations.

The American Diabetes Association (ADA) guidelines recommend 150 min per week of moderate intensity activity to help manage diabetes [7], approximating around 7000 steps per day [8]. Those at risk of foot ulceration must try to undertake sufficient physical activity to maintain glycaemic control [9, 10] and cardiovascular health [11] whilst also avoiding excessive trauma to their feet. Striking the balance between insufficient and excessive physical activity is key. Being inactive is defined as an insufficient amount of physical activity during waking hours characterised by activity below a specified threshold [12]. Sedentary behaviour is defined as any waking behaviour characterised by an energy expenditure below a specified threshold (such as a metabolic equivalent threshold [MET] e.g.) whilst in a sitting or reclining posture [13]. It is possible to be both active and sedentary, that is, meet physical activity guidelines, but also spend a lot of time sedentary. Figure 1 illustrates the possible permutations.

In a systematic review, Tao et al. [14] concluded that being inactive (less than 150 min of weekly exercise) was a significant predictor of the first ever instance of diabetes related foot ulcers (OR 12.1, *p* < 0.01). However, this conclusion was based on only two studies (*n* = 175 and 323) and sedentary behaviour was not considered in relation to DFU risk. Similarly, in their systematic review of predictive factors for DFU, Monteiro‐Soares et al. [15] reported that being inactive was directly associated with higher risk of DFU (3 studies, see Supporting Information S1: Table S1). Again, sedentary behaviour as distinct from being inactive was not considered.

Finally, Wondmeneh et al. [16] suggested that being physically inactive more than doubled DFU risk (aOR = 2.26, 95% CI: 1.04–3.48, *p* < 0.05). However, their systematic review evidence was limited to three studies. In each instance, the studies referred to by each systematic review were different (Supporting Information S1: Table S1) which strongly suggests that a further systematic review is necessary to synthesise the evidence that being physically inactive may be associated with DFU risk, and to examine whether there is a similar relationship between sedentary behaviour and DFU risk.

Previous reviews have been limited to the effect of exercise interventions, and prescribed physical activity [17, 18, 19] (Supporting Information S1: Table S1) or devised‐assessed weight‐bearing physical activity [20] on risk classifications rather than habitual physical activity and sedentary behaviour on the prospective risk of DFU. This is an important distinction as people with diabetes who have had or currently have foot ulcers are known to decrease their activity levels [20] and are advised to offload [21].

The aims of this systematic review are: (1) To determine whether physical inactivity and/or sedentary behaviour are associated with increased risk of DFU and (2) To determine whether there is consensus with regard to a threshold for minimum physical activity and/or sedentary behaviour required to reduce the risk of DFU.

## 2 Methodology

This systematic review was performed using the Preferred Reporting Items for Systematic Reviews and Meta‐Analyses (PRISMA) guidelines [22] and both the protocol and search criteria were registered on the PROSPERO database (reference no. CRD42021286813).

### 2.1 Eligibility Criteria

We sought peer‐reviewed studies published in the English language involving people with diabetes (type 1 or 2) which reported their physical activity and/or sedentary behaviour (including both survey‐based and objectively measured activity) and foot ulceration outcomes. Case reports and studies with proscribed physical activity interventions as opposed to observational studies of habitual physical activity and/or sedentary behaviour were excluded along with studies reporting physical activity for different classifications of foot ulcer risk rather than foot ulcer outcomes.

### 2.2 Data Sources and Search Criteria

Medline, Scopus and Embase databases were searched until 4 January 2026 without starting date restriction for studies as described above using the search criteria of (‘diabetes’ OR ‘diabetic’) AND (‘physical*’ OR ‘activ*’ OR ‘inactiv*’ OR ‘sedentary’) AND ‘foot’ AND ‘ulcer’.

### 2.3 Study Selection

Two reviewers (P.J.J. and A.N.) independently reviewed the papers to determine which papers should be shortlisted, each blinded to the other's shortlist. A third reviewer (G.T.) compiled the final list for inclusion, which was then reviewed and discussed by the entire group of authors (A.N., A.V.R., D.G.A., G.T., H.S., P.J.J.) and the inclusion list finalised.

### 2.4 Risk of Bias Assessment

Study risk of bias was independently assessed by two researchers (A.N. and P.J.J.) using the Newcastle‐Ottawa Scale (NOS) blinded to each other's assessments [23, 24]. Disagreements were resolved by a third reviewer (G.T.).

### 2.5 Exploratory Meta‐Analysis

The exploratory meta‐analysis was performed using MetaAnalysisOnline.com (A5 Genetics Ltd., Budapest, Hungary) [25]. Studies were included if they reported an odds ratio estimate and 95% confidence interval for the relationship between physical inactivity and diabetes‐related foot ulceration or lower limb ulceration. A primary exploratory analysis included studies where the physical activity threshold was reported. A wider exploratory analysis also included studies where physical inactivity or lack of regular exercise was reported, but the amount or intensity of physical activity was not clearly defined. Pooled odds ratio estimates were interpreted cautiously because the included studies differed in design, exposure definition and outcome definition, and because adjusted and unadjusted odds ratio estimates were combined.

## 3 Results

### 3.1 Study Selection

**FIGURE 2:**
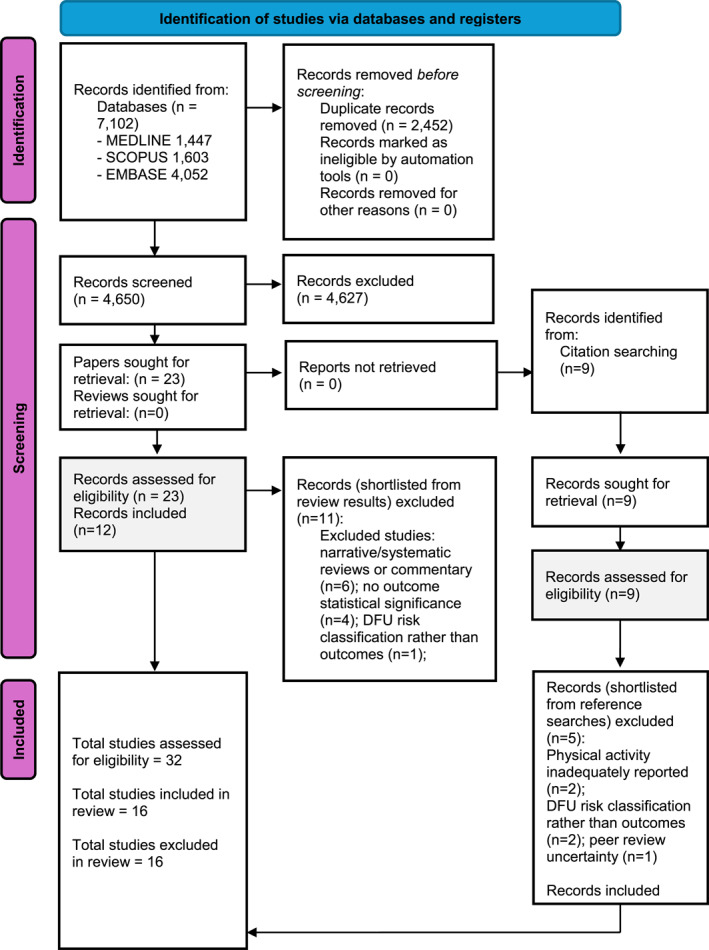
PRISMA diagram.

A total of 7102 studies were identified from which 2452 duplicates were excluded and 4627 were excluded based on the title or abstract (Figure 2). Two reviewers (P.J.J. and A.N.) then independently shortlisted papers, which were reviewed by a third reviewer (G.T.) to produce the final shortlist for inclusion, which was circulated to all authors for comment (P.J.J., A.N., G.T., D.G.A., H.S., A.V.R.). Twenty‐three papers were assessed for eligibility, plus an additional nine papers identified from reference searching. Sixteen papers in total were included, with 16 excluded where physical activity was inadequately reported (*n* = 2), ulcer outcomes were unreported (*n* = 3) or reported no statistically significant association (*n* = 4), peer review uncertainty (*n* = 1) or were not studies (*n* = 6).

### 3.2 Study Size, Proportion With DFU and Geographical Location

The included studies involving habitual physical activity and DFU outcomes ranged in size from 100 [26] to 1751 participants [27] (Table 1). There were five cross‐sectional studies [27, 32, 35, 36, 38], five case‐control studies [28, 30, 34, 37, 39], and six cohort studies [26, 29, 31, 33, 40, 41]. One of these cohort studies assessed physical activity within 400 participants previously split into three groups for a randomised controlled trial evaluating two types of therapeutic footwear and inserts against usual footwear [41]. Among these included study participants, 7.4% [27] to 50.0% [39] of participants ulcerated (*n* = 30–162 [27, 39]). Sixty‐two percent of these studies were carried out either in Africa [28, 32, 34, 35, 36, 38] or the Middle East [29, 30, 33, 39] with Ethiopia [28, 32, 34, 35, 36, 38] and Iran [29, 33, 39] accounting for over half (56.2%, Supporting Information S1: Figure S1).

**TABLE 1:** Physical inactivity and diabetes‐related foot ulceration.

Lead author (year)	Size	%DFU	Physical activity targets		Strength of relationship of physical inactivity with DFU
			Target quantity (minutes per week)	Target intensity	
Tola [28] (2021)	502	21.0%	210 min per week (30 min per day)	Moderate‐vigorous (e.g. field working)	47.2% DFU inactive versus 20.5% non‐DFU inactive. aOR 2.29, *p* < 0.05
Yazdanpanah [29] (2018)^a^	566	5.3%	150 min per week (3 days per week)	Moderate‐vigorous	Physical activity relationship with 2‐year DFU‐free survival rate: HR 1.52 (95% CI: 0.65–3.54), *p* = 0.33
Badedi [30] (2019)	323	33.4%	150 min per week	Moderate (e.g. brisk walking)	94.4% DFU inactive versus 66.0% non‐DFU inactive, *p* < 0.001
Orlando [31] (2021)	175	35.5%	150 min per week	Moderate (aerobic)^b^	1.7% DFU active versus 34.5% non‐DFU active, *p* = 0.001
Mekonen [32] (2024)	332	13.0%	150 min per week (30 min per day)	Unknown	20.9% DFU inactive versus 20.4% non‐DFU inactive, *p* = 0.94
Yazdanpanah [33] (2024)	901	8.0%	90–120 min per week (30 min alt. days)	Moderate (e.g. walk, swim, cycle, no threshold) ^b^	83.3% DFU inactive versus 68.8% non‐DFU aOR 2.25 (95% CI: 0.95–5.35), *p* = 0.066
Woldemariam [34] (2020)	161	32.9%	90 min per week (30 min × 3 times)	Moderate‐vigorous (e.g. walking, jogging or running)	39.6% DFU inactive versus 29.6% non‐DFU inactive, aOR 1.52 (95% CI: 0.58–4.02), *p* = 0.40
Molvær [27] (2014)	1751	7.4%	60 min per week	Any intensity	41.4% DFU inactive versus 23.2% non‐DFU inactive, p < 0.001
Abdissa [35] (2020)	277	11.6%	Unknown	Unknown	DFU: 68.8% versus non‐DFU: 31.2%, *p* = 0.21 COR 1.6 (95% CI: 0.7–3.6)
Hirpa [36] (2023)	278	18.7%	‘Physical activity’	Unknown	aOR 1.87, (95% CI: 0.7–4.7), *p* = 0.18
Jahan [37] (2023)	196	33.2%	No target quantity^b^	No target intensity^b^	OR 3.43 (95% CI: 1.7–7.1), *p* = 0.001
Negash [38] (2022)	267	11.2%	‘Regular exercise’	Unknown	aOR 3.91, (95% CI: 1.5–10.1), *p* = 0.003
Bagheri [39] (2025)	800	50.0%	600‐1500 MET per week	Moderate (< 600 MET low) (> 1500 MET high)	DFU: 11% low MET versus non‐DFU: 2%; 69% high MET versus 91%, *p* < 0.001. High activity (> 1500 MET) protective: OR 0.12, *p* < 0.001
Orlando [31] (2021)	175	35.5%	8 h per day (‘sedentary time’)	≤ 1.5 MET	DFU 12.8 h/day ± 3.0 versus non‐DFU: 9.4 ± 3.1, *p* = 0.004; OR 2.95 (95% CI: 1.5–6.4), *p* = 0.008^c^
Armstrong [26] (2004)	100	8.0%	N/A	N/A	Average daily steps: 809.0 ± 612.2 DFU versus 1394.5 ± 868.5 non‐DFU, *p* = 0.03 Coefficient of variation in daily steps: 96.4 ± 50.3 DFU versus 44.7 ± 15.4 non‐DFU, *p* = 0.0001 Coefficient of variation 2 weeks prior to DFU: 115.4 ± 43.0, *p* = 0.02
LeMaster [26] (2008)	400	15.5%	≥ 7.5 active hours per day	Active hour = 60 min standing, walking etc.	‘Most active’ participants were at significantly reduced risk of ulceration OR 0.20 (95% CI: 0.0–0.9), *p* ≤ 0.05
Waaijman [40] (2014)	171	41.5%	N/A	N/A	Average daily strides: 3238 ± 1287 DFU versus 3437 ± 1990 non‐DFU, OR 0.99 (95% CI:0.97–1.01), *p* = 0.40
					Variation in daily strides OR 0.91 (95% CI: 0.9–1.0), *p* = 0.01

*Note:* Target quantity: Green background indicates statistically significant, red indicates not statistically significant. Strength of relationship of physical activity with ulceration: aOR = Adjusted Odds Ratio, CI = Confidence interval, HR = Hazard Ratio; OR = Odds Ratio.

Abbreviations: DFU = Diabetes‐related foot ulceration. N/A = Not applicable.

^a^ Consisting of at least 3 days per week with no more than two consecutive days without activity.

^b^ Indicates marked data obtained through author enquiry.

^c^ Risk of sedentary behaviour independent of physical activity status.

### 3.3 Physical Activity, Sedentary Behaviour and DFU Outcomes

Eleven of 16 (69%) studies reported significantly greater likelihood of foot ulceration in participants who were classified as inactive. The direction of the association was broadly consistent in the remaining studies, albeit non‐significant. The study methodologies varied considerably.

Only one of the included studies [31] assessed sedentary behaviour as distinct from physical inactivity (Figure 1) where 8 hours per day of sedentary time ≤ 1.5 MET was associated with an almost tripled risk of diabetes‐related foot ulceration (OR 2.95, 95% CI: 1.5–6.4, *p* = 0.008, see Table 1).

### 3.4 Exploratory Analysis of Physical Inactivity and DFU Risk

**FIGURE 3:**
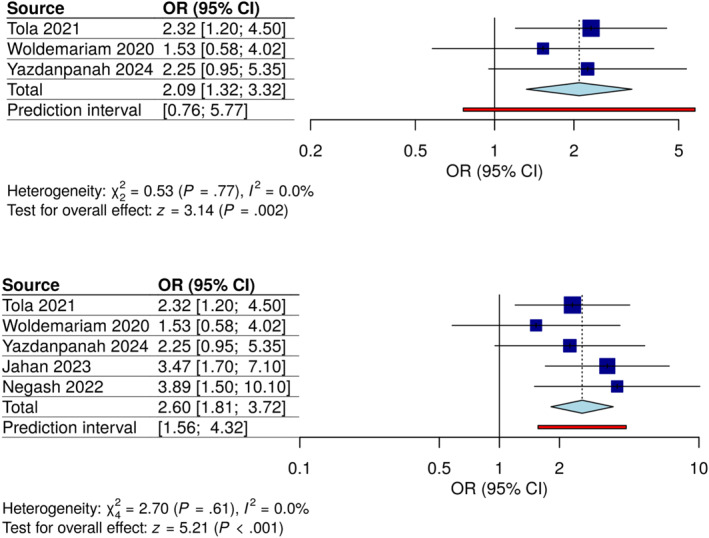
Exploratory analysis of the relationship between physical activity and DFU or lower limb ulceration. Primary analysis: Studies where the physical activity threshold, Odds Ratio (OR) and confidence interval were reported. Secondary analysis: Wider exploratory analysis where the physical activity threshold was unreported.

Figure 3 shows an exploratory meta‐analysis of studies reporting odds ratio estimates for physical inactivity and diabetes‐related foot ulceration. In the analysis including Tola et al. [28], Woldemariam et al. [34], and Yazdanpanah et al. [33], physical inactivity (defined variously as moderate‐vigorous or moderate activity, for 210, 90 or 920 min respectively) was associated with higher odds of DFU or lower limb ulceration. The pooled OR was 2.09, 95% CI 1.32–3.32, *p* = 0.002. The prediction interval was wider and crossed one: 0.76–5.77. There was no evidence of statistical heterogeneity, although only three studies were included: *χ* ^2^_2_ = 0.53, *p* = 0.77, and *I* ^2^ = 0.0%. A wider exploratory analysis added Jahan et al. [37] and Negash et al. [38]. These studies reported a significant association between physical inactivity or lack of regular exercise and DFU, but did not clearly define the amount or intensity of physical activity. With these studies included, the pooled OR was 2.60, 95% CI 1.81–3.72, *p* < 0.001. The prediction interval was 1.56–4.32. Again, there was no evidence of statistical heterogeneity: *χ* ^2^_4_ = 2.70, *p* = 0.61; *I* ^2^ = 0.0%. This larger estimate should be interpreted cautiously because the added studies had less clear physical activity definitions.

### 3.5 Evidence Towards the Protective Quality of Physical Activity in DFU Risk

In Bagheri et al. [39], physical activity with a Metabolic Equivalent of Task (MET) of more than 1500 MET per week was slightly protective (OR 0.1, *p* < 0.001) [39]. Similarly, those engaged in 7.5 active hours or more of standing or walking each day were slightly less likely to ulcerate (OR 0.2, *p* ≤ 0.05 [40]).

### 3.6 Habitual Physical Activity Measurement Method

Only two studies measured habitual physical activity using devices (e.g., step activity monitors, accelerometers etc.) in relation to diabetes‐related foot ulcer outcomes [26, 41]. Armstrong et al. [26] used a waist‐mounted accelerometer/pedometer worn for a minimum of 25 weeks (37.1 ± 12.3 weeks) or until ulceration [26] while Waaijman [41] used an ankle‐worn step activity monitor worn for a 7‐day period [41] (Supporting Information S1: Table S2). The former study measured physical activity in steps [26] and the latter in strides where a stride was defined as the heel‐strike of one foot to the heel‐strike on the next successive step of the same foot (i.e., two steps) [41].

Continuous physical activity monitoring in Armstrong et al. [26] found lower average daily steps in those who ulcerated during a 25‐week follow‐up than those who did not (809 ± 612 steps vs.1394 ± 868, *p* = 0.03, Risk of bias [ROB]: poor).

In Waaijman et al. [41], average daily strides were lower in those who ulcerated than those who did not (3238 ± 1287 vs. 3437 strides ± 1990, i.e., 6476 ± 2574 vs. 6874 ± 3980 steps, ROB: fair), but this difference was not statistically significant.

Two studies (12.5%) found that variation in device‐assessed total daily steps was related to ulceration [26, 41]. However, they differ as to whether variation in steps increases or decreases DFU risk. In Armstrong et al., the coefficient of variation was significantly greater in those ulcerating than those without ulcers (96.4% ± 50.3 vs. 44.7% ± 15.4, *p* = 0.0001), with variability increasing further in the fortnight prior to ulceration (115.4% ± 43.0 *p* = 0.02) [26]. In contrast, Waaijman et al. found that increased day‐to‐day variation in daily stride count was associated with lower odds for ulcer recurrence (odds ratio: 0.91, 95% CI 0.86–0.96) *p* = 0.001 [41].

### 3.7 Self‐Reported Assessment of Habitual Physical Activity

The remaining studies (*n* = 14, 87.5%) measured habitual physical activity through interviews (*n* = 1) [37], interview‐based questionnaires (*n* = 3) [35, 36, 38], questionnaires (*n* = 9) [27, 29, 30, 31, 32, 33, 34, 39, 40] or patient records (*n* = 1) [28] (Supporting Information S1: Table S2).

Half of all questionnaires were developed in‐house without clear validation (*n* = 8 [29, 30, 33, 34, 35, 36, 38, 40], Supporting Information S1: Table S3) with four tested prior to use [34, 35, 38, 40]. However, the number of participants in questionnaire test uses was small (*n* = 8 [34]) or unreported [35, 38, 40]. Three studies used pre‐existing questionnaires to evaluate physical activity [31, 32, 39], including the International Physical Activity Questionnaire (IPAQ) short form [42], Physical Activity Scale (PAS 2.1) [43], and 7‐day Physical Activity Recall (PAR) [44] (Supporting Information S1: Table S3).

Survey‐obtained habitual physical activity data was either based on the previous day's physical activity (assessed every 17 weeks) [40], previous week's physical activity [31, 39] or perceived average day [28] or week [27, 32, 34] (Supporting Information S1: Table S2). In other studies, this was unreported [29, 30, 33, 35, 36, 37, 38]. It is important to note that self‐report and device‐assessed physical activity are conceptually distinct [45]; outcomes are not interchangeable and often correlate poorly.

Self‐reported physical activity studies classified participants as active or inactive. The most common threshold for physical inactivity was below 150 min per week of moderate intensity physical activity [29, 30, 31, 32] (Table 1), explicitly derived in three studies [29, 30, 31] from the American Diabetes Association Standards of Medical Care in Diabetes (2016–2019) [46, 47, 48].

In Badedi [30] 94.4% of those with DFU were physically inactive as compared with 66.0% of those without DFU (*p* < 0.001, *n* = 323 study size). In Orlando, only 1.7% of those with DFU were active based on this threshold as compared with 34.5% of those without DFU (*p* = 0.001, *n* = 175 [31]). However, in the larger study of Yazdanpanah (*n* = 566) physical inactivity was not a statistically significant factor in ulceration outcomes (*p* = 0.33, [28]) and similarly in Mekonnen and Gebru [32], inactivity levels in both those with and without DFU were very similar (20.9% vs. 20.4%, *p* = 0.94, *n* = 332) [32].

Studies utilising other classifications of physical activity status did not provide a justification [28, 33, 34]. Tola et al. [28] reported that not engaging in moderate‐vigorous intensity for 210 min per week doubled the risk of DFU (aOR 2.3, 95% CI: 1.2–4.5, *p* < 0.05, ROB: Fair, Table 2). However, outcomes included either foot or lower limb ulceration. Other thresholds such as 90–120 min per week of moderate physical activity [33] or 90 min per week of moderate to vigorous physical activity [34] were not statistically significant with regard to DFU outcomes (Table 1). An exception was a large study (*n* = 1751) which found that 41.4% of those with DFU did not engage in 60 min per week of physical activity at any intensity [34] as compared with 23.2% of those without ulceration (*p* < 0.001, Table 1).

**TABLE 2:** Newcastle–Ottawa Scale risk of bias analysis.

Case‐control studies								
Lead author	Adequate case definition	Case representativeness	Control selection	Definition of controls	Comparability of cases and controls	Ascertainment of exposure	Non‐response rate	Quality
Badedi [30]	0	0	0	*	0	0	0	POOR
Bagheri [39]	0	*	0	*	**	0	0	FAIR
Jahan [37]	0	0	0	0	0	*	0	POOR
Tola [28]	*	0	0	0	**	*	0	FAIR
Woldemariam [34]	0	*	0	0	0	0	0	POOR

Cohort studies									
Lead author	Representativeness of exposed cohort	Selection of non‐exposed cohort	Ascertainment of exposure	Outcomes of interest	Comparability of cohorts	Assessment of outcome	Follow‐up long enough	Adequacy of follow‐up	Quality
Armstrong [26]	*	0	*	0	0	0	*	0	POOR
Orlando [31]	*	0	0	0	**	0	*	0	POOR
LeMaster [40]	0	0	0	*	**	*	*	0	POOR
Waaijman [41]	0	0	*	*	**	*	*	0	FAIR
Yazdanpanah [29]	*	0	0	*	**	0	*	0	POOR
Yazdanpanah [33]	*	0	0	*	**	0	*	0	POOR

Cross‐sectional studies (NOS‐X)							
Lead author	Representativeness of study sample	Sample size	Ascertainment of exposure	Assessment of outcome	Adjustment for confounder	Assessment of confounders	Quality
Abdissa [34]	*	*	*	0	**	*	LOW
Hirpa [27]	*	*	0	0	**	*	LOW
Mekonen [30]	*	*	0	0	**	*	LOW
Molvær [33]	*	*	0	0	**	*	LOW
Negash [36]	0	*	*	*	**	*	LOW

*Note:* 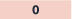, 0 points; 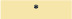, 1 points; 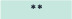, 2 points. Overall rating as report: 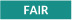, 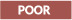, 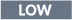.

Whilst there was evidence from other studies that physical inactivity could triple the risk of DFU: OR 3.4 (95% CI: 1.7–7.1), *p* = 0.001 [37] and aOR 3.9 (95% CI: 1.5–10.1), *p* = 0.003 [38] the target quantity of physical activity and its intensity were undefined severely limiting their application [37, 38] (ROB: poor, Table 2). In other studies, thresholds for physical activity and its intensity were unreported and not significant in relation to DFU outcomes [35, 36].

### 3.8 Diabetes‐Related Foot Ulcer Outcomes Within Physical Activity Studies

Four studies concerned diabetes‐related foot ulcers expressly excluding ulcers located at the ankles (*n* = 4 [31, 32, 35, 36], Supporting Information S1: Table S3), one included foot or ankle ulcers [39] and another lower limbs ulcers more generally [28]. Other studies either did not define foot ulcers [26, 27, 29, 33, 34, 37, 38, 40] or referred to new or poorly healing or non‐healing ulcers [30] rather than necessarily new ones or recurrent ulcers (unclear whether these were necessarily at the same site) [41].

Ulcer severity, where defined, either included both partial and full thickness lesions of the skin [30, 32] or only full thickness lesions [31, 35, 41]. In other studies, ulcer severity was undefined or included ulceration, infection, or deep tissue involvement [28].

### 3.9 Risk of Bias Within Habitual Physical Activity Studies With DFU Outcomes

Among the 16 studies included in this systematic review, three were rated as fair [28, 39, 41] using the Newcastle–Ottawa Scale risk of bias analysis and 13 studies as poor [26, 27, 29, 30, 31, 32, 33, 34, 35, 36, 37, 38, 40]. Two of the five case control studies (Table 2) were rated as fair involving assessment of physical activity via questionnaire (high activity > 1500 MET per week as DFU protective OR 0.12, *p* < 0.001) [39] and patient records (physical inactivity aOR 2.3, *p* < 0.05) [28]. Of the 6 cohort studies (Table 2), only one study was rated as fair involving objective measurement of physical activity using an ankle step activity monitor worn for 7 days (≥ 7.5 active hours of standing and walking per day where most active at reduced DFU risk OR 0.2, *p* ≤ 0.05) [41].

### 3.10 Type of Diabetes Within Habitual Physical Activity Studies

Four of these studies exclusively involved people with type 2 diabetes mellitus [28, 30, 35, 37] and where reported (*n* = 8, [29, 31, 32, 34, 36, 38, 39, 41]) the percentage of those with type 2 diabetes mellitus ranged from 71.6% [36] to 97.5% [29] (Supporting Information S1: Table S4). The percentage of participants with type 1 or 2 diabetes was unreported in four studies [26, 27, 33, 40].

### 3.11 Demographics Within Habitual Physical Activity Studies

The percentage of female participants ranged from 5.0% [26] to 58.5% [33] (mean: 41.0% ± 14.4, Supporting Information S1: Table S5). The average age of study participants was reported in 11 studies [26, 27, 29, 31, 33, 34, 37, 38, 39, 40, 41]. In other studies, age ranges were reported [28, 32, 35, 36, 38] with < 30 years old accounting for 9.8% [28] to 20.9% [36] and ≥ 50 years old from 34.9% [36] to 53.8% [35]. Average body mass index (BMI) of participants ranged from 27.9 ± 4.7 [39] to 30.7 ± 5.7 [41], as reported in seven studies [26, 27, 29, 31, 33, 39, 41] (Supporting Information S1: Table S6). In the remaining studies BMI was either unreported (*n* = 3, [28, 37, 40]) or classified as underweight (< 18.5 kg/m^2^ [30, 34, 35, 38]) ranging from 1.2% [30] to 10.5% [35]; normal BMI (18.5–24.9 kg/m^2^ [30, 32, 34, 35, 36, 38]) ranging from 26.1% [34] to 82.8% [32]; overweight (25.0–29.9 kg/m^2^ [30, 32, 34, 35, 36, 38]) ranging from 24.2% [32] to 42.1% [30] or obese (≥ 30.0 kg/m^2^ [30, 32, 34, 35, 36, 38] from 3.0% [32] to 38.1% [30] of study participants (Supporting Information S1: Table S6).

Average duration of diabetes for participants within these habitual physical activity studies was reported in 9 studies [26, 27, 28, 29, 31, 33, 34, 39, 41] and ranged from 8.9 ± 6.9 [29] to 21.6 ± 9.1 years [31]. In other studies duration of diabetes was either unreported [37, 40] or duration of diabetes was classified into year ranges (*n* = 5, [30, 32, 35, 36, 38]) as less than 5 years (6.2% [30] to 58.3% [36]); 5–10 years (19.9% [38] to 31.3% [30]) or more than or equal to 10 years (13.0% [32] to 62.5% [30], Supporting Information S1: Table S7).

Ethnicity of participants within these habitual physical activity studies was unreported in the majority of studies (12 of 16, [26, 27, 28, 30, 32, 34, 35, 37, 38, 39, 40, 41]). Four studies [29, 31, 33, 36] reported the ethnicity of study participants. These studies described Ethiopian [36] and Iranian ethnic groups [29, 33] participating in the study, with the remaining study entirely comprised of Caucasian participants substantially limiting its applicability [31] (Supporting Information S1: Table S8).

Loss of protective sensation associated with neuropathy was assessed primarily through either the Michigan Neuropathy Screening Instrument (MNSI) questionnaire typically involving an exam question score ≥ 2.5 (Ref. [32, 35, 36], Supporting Information S1: Table S9) or a 10‐g monofilament [30, 34, 39, 40, 41] combined with a neurothesiometer to assess Vibration Perception Threshold (VPT) [41] or tuning fork and temperature sensing examination [30] in some instances. Vibration perception threshold alone [29] or electromyography of the peroneal motor never and sural sensory nerve [31] were also utilised. In all other instances, loss of protective sensation testing methodology was unreported (*n* = 5, [27, 28, 33, 37, 38]). Detailed methodological information such as VPT threshold [29], frequency of tuning form (i.e., 128 or 256 Hz) [30], or number and location of monofilament sites [30, 34, 39, 40, 41] were often unreported.

Foot deformity was unreported in 10 of the 16 studies [26, 27, 29, 30, 32, 33, 38, 39, 40]. Foot deformities found within participants included hammer toes [31, 35, 36, 41], claw toes [31, 34, 35, 36, 41], prominent metatarsal heads [31, 35, 36, 37, 41], amputations [31, 35, 36, 37, 41], and hallux valgus [34, 35, 36, 41] (Supporting Information S1: Table S10). Participants were also assessed for pes cavus [31, 41], pes planus [41] and pes equinus [41].

Where defined, the percentage of habitual physical activity study participants with foot deformities ranged from 33.1% [31] to 90.1% [41].

The type of footwear (i.e., therapeutic or non‐therapeutic) worn by people with diabetes during habitual physical activity with DFU outcomes was frequently unreported (*n* = 12, [27, 28, 29, 30, 32, 33, 34, 35, 36, 37, 38, 39], Supporting Information S1: Table S11). All participants wore therapeutic footwear in two studies [26, 41] and 36.6% [31] or 50.0% in the others. Footwear fit was assessed in just two of these studies [33, 34].

## 4 Discussion

This review considered all available evidence on whether physical inactivity or sedentary behaviour is potentially associated with diabetes‐related foot ulcers.

In contrast, previous reviews have focused on device‐based assessment only [20], the effect of exercise interventions, or prescribed physical activity [17, 18, 19]. Studies broadly suggested that being inactive and/or sedentary may increase the risk of DFU. However, considerable heterogeneity in methods used to measure and define exposure limits the determination of recommended amounts of physical activity and/or sedentary behaviour. To enable progress towards this aim, we discuss the implications of this heterogeneity, explore possible mechanisms, then make several recommendations for future research. These include (1) the need for geographical diversity and reporting of ethnicity of study participants; (2) clear definitions of physical activity exposures and ulcer outcomes; and (3) utilisation of the full potential of physical activity metrics (activity type, intensity and device‐derived features) to facilitate development of thresholds customised to individuals.

### 4.1 Difficulties of Deriving a Physical Inactivity and Sedentary Behaviour Threshold to Improve DFU Outcomes

Four studies suggested the 150 min per week of moderate to moderate‐vigorous physical activity recommended by the American Diabetes Association Standards of Diabetes Medical Care as a threshold below which constituted physical inactivity [28, 29, 30, 31]. While this classification of active/inactive physical activity was statistically significant in its association with DFU outcomes in two of these four studies (*n* = 175–323 [30, 31]), it was not significant in other larger studies (*n* = 332–566 [29, 30]). This classification is also consistent with the World Health Organisation classification of habitual physical activity as active and inactive [49].

In a large study (*n* = 502) those below a higher threshold of 210 min per week of moderate to vigorous activity had over double the risk of foot or lower limb ulceration (aOR 2.3, *p* < 0.05, NOS rating: fair). However, the reasoning or source of this threshold is unreported. Further studies are required to confirm these findings given that the study population was confined to people with type 2 diabetes and wound sites were unreported, which could include lower extremity ulcers as well as feet. In two further studies [37, 38], being inactive was reported to triple DFU risk (OR 3.4, *p* = 0.001; aOR 3.9, *p* = 0.003), but thresholds for both target quantity and intensity of physical activity were unreported and risk of bias was high.

Only one study considered sedentary time, and reported that spending 8 h per day in sedentary time (≤ 1.5 MET) [31] almost tripled DFU risk (OR 2.95, *p* = 0.008). This should be interpreted with caution as this was based on a single prospective cohort (*n* = 175) and was assessed as having a high risk of bias (NOS rating: poor).

### 4.2 Exploratory Analysis of Physical Inactivity and DFU Risk

The exploratory analysis was in keeping with the main findings of this review. When only the three studies with more clearly reported physical activity thresholds were included [28, 33, 34], physical inactivity was associated with around twice the odds of DFU or lower limb ulceration; however, the prediction interval crossed one. When two further studies were added [37, 38], the pooled estimate increased. However, these studies did not clearly define the amount or intensity of physical activity, which makes the result harder to apply clinically. This wider exploratory analysis should therefore be interpreted with caution. Taken together, the exploratory analysis suggests that physical inactivity may be linked with higher DFU risk. However, the evidence remains limited by the small number of studies, different definitions of physical inactivity, the mixing of adjusted and unadjusted odds ratio estimates, and poor risk‐of‐bias ratings in several included studies.

### 4.3 How Could Physical Inactivity or Sedentary Behaviour Theoretically Increase DFU Risk?

It is possible that lower step counts might lead to tissue atrophy and weakness leading in turn to ulcer development even at relatively low levels of daily cumulative stress [26]. The role of physical inactivity and sedentary behaviour upon intrinsic muscle sarcopenia [50], glycaemic control (including glycaemic control lowering wound‐healing readiness [51]) and cardiovascular health [52] is well known. Sedentary behaviour is also associated with ischaemia‐reperfusion injury [53], endothelial dysfunction and microcirculatory impairment [54]. It is also possible that plantar skin health and elasticity are adversely affected by seated weight‐bearing forms of sedentary behaviour [55].

### 4.4 Variation in Daily Steps: Friend or Foe?

The effect of variation in daily step counts upon ulcer outcomes is not clear. While two studies agree that variation in daily steps is related to ulceration, the first study [26] found that ulceration was related to a higher coefficient of variation in daily steps, whereas the second [41] found the opposite, that day‐to‐day variation in stride count decreased the risk of ulceration. We note that the risk profile of participants in these two studies differed: all the participants in the second study had a history of ulceration, whereas in the former, only 32% had. The average diabetes duration also differed (17 years [41] vs. 13 [26]) and activity was monitored for at least 25 weeks in the former [26] but just a week in the latter [41]. However, it is unclear the extent to which these differences may explain the differences in results. In either case, these studies illustrate that the role of physical activity in DFU risk is more complex than deriving a goldilocks target between the extremes of too little or too much activity. Spikes or troughs in physical activity may stress tissue that has lost its capacity to adapt with its resiliency subject to individual gait, footwear choices and skin care routines.

### 4.5 Recommendations for Future Research

Fifty‐six percent of the included studies are Ethiopian or Iranian, with 62% from the Middle East or Africa (Saudi Arabia, Iran and Ethiopia). These are populations that substantially differ from those in developed countries in terms of their footwear, occupation (subsistence farming vs. desk work), walking patterns, and healthcare access. Climate may also play a role both in inactivity, particularly during periods when drought or temperature affect exercise and patterns of daily, weekly or seasonal activity. Physical inactivity here may therefore have significant differences from physical inactivity in developed countries. There is a need both for further research within a broader range of countries, and better reporting of ethnicity amongst study participants along with other key factors such as loss of protection of sensation and habitual footwear worn among participants.

Methodological differences included definitions of habitual physical activity (e.g., previous day/week or average day/week) and foot ulcers (e.g., full thickness lesions, full and partial lesions, inclusion of ankle or lower limb wounds as well as foot ulcers etc.). Only two of the 16 included studies device‐assessed physical activity. All other studies were based on deriving habitual physical activity from questionnaires, medical records, or interview‐derived subjective data from study subjects. Newcastle‐Ottawa Scale assessed risk of bias was high for the majority of the included studies. Cross‐sectional and case‐control designs may have difficulty in distinguishing inactivity causes of DFU as distinct from prodromal DFU or the neuropathy and related proprioception issues and deformity preceding it, which themselves may cause inactivity.

Assessing the impact of inactivity and sedentary behaviour upon diabetes‐related foot ulcer outcomes will require prospective cohort studies using consumable wearables to provide a fuller picture of habitual physical activity—for example, time spent engaged in different physical activity types (e.g., walking, standing, cycling, sitting) and utilising accelerometer‐derived features such as dominant frequency, mean and standard deviation of acceleration axes which can facilitate classification of physical activity by type and intensity which have yet to be used in this context. There is also a need for further studies examining the independence and interrelationship of both physical activity and sedentary behaviour upon DFU outcomes.

### 4.6 Strengths and Limitations

The strengths of this study include its extensive literature search strategy with a comparatively large size (4650 records were examined), which has uncovered double the number of questionnaires, interviews, and medical records derived habitual physical activity studies (*n* = 14) than the combined total (*n* = 7) identified in previous reviews (Ref. [14, 15, 17, 19], Supporting Information S1: Table S1). Other strengths include the absence of any start date restriction on searches, blinded independent shortlisting performed by two reviewers and blinded Newcastle–Ottawa Scale study risk of bias assessment. Limitations include the large number of possible alternative keywords which can describe specific aspects of the general categories of habitual physical activity, inactivity, or sedentary behaviour. These include walking, standing, steps, strides, and sitting. Which might easily generate an unmanageable volume of results.

## 5 Conclusions

The available evidence suggests that physical inactivity may increase the risk of diabetes‐related foot ulceration although the available evidence often has a high risk of bias with methodological heterogeneity and key data around ethnicity or neuropathy assessment unreported. Physical inactivity can double foot and lower limb ulceration risk in people with diabetes (aOR 2.3, 95% CI: 1.2–4.5, *p* < 0.05, NOS: fair) based on a target 210 min per week of moderate‐vigorous intensity (1 study, *n* = 512). Other evidence that physical inactivity tripled DFU risk had a high risk of bias and failed to define target quantity and intensity. Evidence that sedentary behaviour increases DFU risk was limited to a single prospective cohort study with a high risk of bias.

With the growing use of wearables and artificial intelligence to process the data they produce, the future is likely to encompass personalised thresholds for activity, inactivity, sedentary behaviour and cumulative plantar stress. It is hoped that this systematic review will act as a catalyst for further prospective cohort studies involving wearables to assess the impact of physical inactivity and sedentary behaviour in DFU outcomes and the development of personalised activity targets.

## Author Contributions

P.J.J. was responsible for the conceptualisation, study design, systematic literature search, original draft of the manuscript and initial synthesis of findings. P.J.J., A.N. and G.T. classified the retrieved information and undertook the formal analysis and data verification, while A.V.R., D.G.A. and H.S. provided intellectual input and critical oversight of the analytical process. G.T. carried out the exploratory meta‐analysis of studies and produced all visualisations (figures and images). A.N., A.V.R., D.G.A., G.T. and H.S. critically reviewed and edited the manuscript. All authors have read and approved the final version of the manuscript.

## Funding

A.V.R. is supported by the National Institute for Health Research (NIHR) Leicester Biomedical Research Centre and NIHR Applied Research Collaboration East Midlands (ARC‐EM). The funders had no role in the design and conduct of the study; collection, management, analysis, and interpretation of the data; preparation, review, or approval of the manuscript; and decision to submit the manuscript for publication. For the purpose of open access, the author has applied a Creative Commons Attribution licence (CC BY) to any Author Accepted Manuscript version arising from this submission. P.J.J.'s contribution to this research was funded by the National Institute for Health and Care Research (NIHR) BRC Leicester (NIHR203327). The views expressed are those of the author(s) and not necessarily those of the NIHR or the Department of Health and Social Care. P.J.J. would also like to thank the Anglo‐Austrian Society Otto Harpner fund for a visit to H.S. at Medical University of Graz in November 2024 which led to this project. D.G.A.'s contribution to this study is partially supported by National Institutes of Health, National Institute of Diabetes and Digestive and Kidney Diseases Award Number 1R01124789‐01A1.

## Conflicts of Interest

A.N., A.V.R., G.T., H.S., P.J.J.—no conflicts of interest to declare. D.G.A.: This study is partially supported by National Institutes of Health, National Institute of Diabetes and Digestive and Kidney Diseases Award Number 1R01124789‐01A1.

## Supporting information


Supporting Information S1

## Data Availability

The data that support the findings of this study are available from the corresponding author upon reasonable request.
