# Supplementary material for: Less Is More? Physical Inactivity and Increased Risk of Diabetes‐Related Foot Ulceration—A Systematic Review
Source: Diabetes Metab Res Rev. 2026 Jul 6;42(5):e70198. doi: 10.1002/dmrr.70198 (PMC13338649; doi:10.1002/dmrr.70198)
Supplement: Supplementary file 1 — Supporting Information S1 [file DMRR-42-e70198-s001.docx]

**Supp. Table 1 Systematic reviews of DFU risk factors featuring physical activity (PA)**

| **Lead author (year)** | **No. of PA studies  included** | **Relationship between habitual physical activity and diabetes-related foot ulcer outcomes** | **Habitual PA studies included (related to DFU outcomes)** |
| --- | --- | --- | --- |
| Crews  (2016) [S1] | 4 | Not applicable – proscribed rather than habitual physical activity | Not applicable |
| Francis  (2019) [S2] | -- | Not applicable – proscribed rather than habitual physical activity | Not applicable |
| Liao  (2019) [S3] | 18 | Not applicable – proscribed rather than habitual physical activity | Not applicable |
| Monteiro-Soares (2012) [S4] | 1  3 | 37.2% DFU inactive vs 27.5% non-DFU inactive, p=0.026. No odds ratio reported.  Smaller average daily activity indicated higher risk | Iversen [S8]  Armstrong [39] LeMaster [40] Maluf [S9] |
| Tao  (2025) [S5] | 2 | Insufficient physical activity (<150 minutes weekly activity) ≃ first-ever DFU  OR 12.6, p<0.01 | Badedi [28], Orlando [29] |
| Van Netten  (2022) [S6] | 8 | Mean steps/day in people with IWGDF risk 3: 6167 (8 studies; 291 participants; weighted mean: 6239 | Armstrong [39] LeMaster [40] Maluf [S9] Waaijman [41] |
| Wondmeneh  (2025) [S7] | 3 | Physical inactivity ≃ DFU aOR 2.3 (95% CI 1.0-3.5), p=0.69* *Reported as statistically significant despite stated p-value | Hirpa [35], Negash [37],  Tola [26] |

**KEY**
aOR = Adjusted Odds Ratio; CI = Confidence Interval; DFU = Diabetes-related foot ulceration; PA = Physical Activity;

**Supp. Table 1 References**

[S1] Crews RT, Schneider KL, Yalla SV, Reeves ND, Vileikyte L, Physiological and psychological challenges of increasing physical activity and exercise in patients at risk of diabetic foot ulcers: a critical review. Diabets Metab./Research & Rev. 2016; 32:791–804. <https://doi.org/10.1002/dmrr.2817>
[S2] Francis P, De Bellis A, Seghieri G, Tedeschi A, Iannone G, Anichini R, Gulisano M, Continuous movement monitoring of daily living activities for prevention of diabetic foot ulcer: A review of literature 2019; Int. J. Prev. Med. 10:22. DOI:10.4103/ijpvm.IJPVM_410_17
[S3] Liao F, An R, Pu F, Burns S, Shen S, Jan Y, Effect of exercise on risk factors of diabetic foot ulcers: A systematic review and meta-analysis. Am J. Phys Med Rehabil. 2019; 98(2):103–116. DOI: 10.1097/PHM.0000000000001002
[S4] Monteiro-Soares M, Boyko EJ, Ribeiro J, Ribeiro I, Dinis-Ribeiro M, Predictive factors for diabetic foot ulceration: A systematic review. DMRR 2012; 28(7):574–600. <https://doi.org/10.1002/dmrr.2319>
[S5] Tao Y, Zhang D, MacGilchrist C, Kirwan E, McIntosh C, Risk factors for first-ever diabetes-related foot ulcer: A systematic review and meta-analysis. Int. Wound J. 2025; 22(8):e70728. <https://doi.org/10.1111/iwj.70728>
[S6] van Netten JJ, Fijen VM, Bus SA, Weight‐bearing physical activity in people with diabetes‐related foot disease: A systematic review. Diabetes Metab Res Rev. 2022;38:e3552. <https://doi.org/10.1002/dmrr.3552>
[S7] Wondmeneh TG, Ebrahim OA, Systematic review and meta-analysis of diabetic foot ulcers and associated factors among adults in Ethiopia. Archives of Public Health 2025; 83:309. <https://doi.org/10.1186/s13690-025-01816-9>
[S8] Iversen MM, Midthjell K, Østbye T, Tell GS, Clipp E, Sloane R et al., History of and factors associated with diabetic foot ulcers in Norway: The Nord-Trondelag Health Study. Scand. J. Public Health 2007; 36(1):62–68. <https://doi.org/10.1177/140349480708531>
[S9] Maluf KS, Mueller MJ, Comparison of physical activity and cumulative plantar tissue stress among subjects with and without diabetes mellitus and a history of recurrent plantar ulcers. Clinical Biomechanics 2003; 18:567–575. <https://doi.org/10.1016/S0268-0033(03)00118-9>

**Supp. Fig. 1** **Geographic distribution of included studies by country**


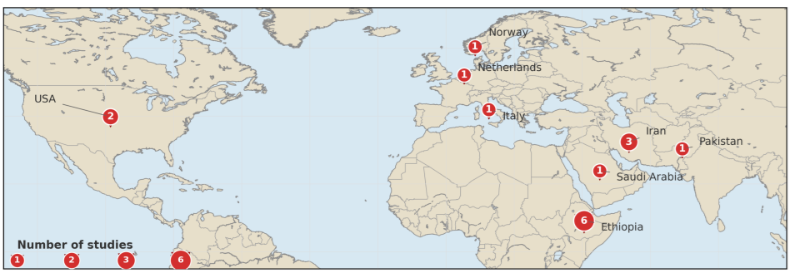


**Supp. Table 2 Study type and methodology including physical activity (PA) period**

|  | **Study** | | | **Physical activity period** |  |  |
| --- | --- | --- | --- | --- | --- | --- |
| **Lead author (year)** | **Type** | **Subtype** | **PA method** |  | **Sample size** | **Country** |
| Abdissa [34] (2020) | Cross-sectional | -- | Interview-based questionnaire | -- | YES (Not PA) | Ethiopia |
| Armstrong [39] (2004) | Observational | Prospective | Device-based | 6 mth (25 wk min) | -- | USA |
| Badedi [28] (2019) | Case-control | Retrospective | Questionnaire | -- | YES (Not PA) | Saudi Arabia |
| Bagheri [38] (2025) | Case-control | -- | Questionnaire | Previous week* | YES (Not PA) | Iran |
| Hirpa [35] (2023) | Cross-sectional | Prospective | Interview-based questionnaire | -- | YES (Not PA) | Ethiopia |
| Jahan [36] (2023) | Case-control | Prospective~ | Interview | -- | YES (Not PA) | Pakistan |
| Orlando [29] (2021) | Cohort | Prospective | Questionnaire | Previous week | NO | Italy |
| LeMaster [40] (2008) | Cohort | Prospective | Questionnaire** | Previous day (every 17 weeks) | -- | USA |
| Mekonen [30] (2024) | Cross-sectional | -- | Questionnaire | Average week | YES (Not PA) | Ethiopia |
| Molvær [33] (2014) | Cross-sectional | -- | Questionnaire | Average week | -- | Norway |
| Negash [37] (2022) | Cross-sectional | -- | Interview-based questionnaire | -- | YES (Not PA) | Ethiopia |
| Tola [26] (2021) | Case-control | Retrospective | Patient records | Average day | YES (Not PA) | Ethiopia |
| Waaijman [41] (2014) | Cohort | Prospective | Device-based | Week (@ 3 mths) | -- | Netherlands |
| Woldemariam [32] (2020) | Case-control | Retrospective | Questionnaire | Average week | YES (Not PA) | Ethiopia |
| Yazdanpanah [27] (2018) | Cohort | Prospective | Questionnaire~ | -- | -- | Iran |
| Yazdanpanah [31] (2024) | Cohort | Prospective | Questionnaire~ | -- | -- | Iran |

**KEY**
--= Unreported. *Inferred through IPAQ questionnaire’s questions. ** Step-activity monitor validation was carried out in a prior study.
Physical activity period: mth = months; wk = week; min = minimum;

**Supp. Table 3 Testing and validation of physical activity questionnaires**

|  | **Questionnaire** | | | |
| --- | --- | --- | --- | --- |
| **Lead author (year)** | **Developed in-house** | **Name (if applicable)** | **Tested prior to use? (n participants)** | **Validated against objective PA?** |
| Abdissa [34] (2020) | YES | -- | YES, unreported. | -- |
| Armstrong [39] (2004) | N/A | N/A | N/A | N/A |
| Badedi [28] (2019) | YES | -- | -- | -- |
| Bagheri [38] (2025) | NO | IPAQ short form [R1] | YES (n=4619) [R1] | No, another form |
| Hirpa [35] (2023) | YES | N/A. Based on WHO Step wise chronic disease risk surveillance [R2] | -- | -- |
| Jahan [36] (2023) | -- | -- | -- | -- |
| Orlando [29] (2021) | NO | Physical Activity Scale (PAS 2.1) [R3] | YES (n=16 HV) [R3] | Unclear |
| LeMaster [40] (2008) | YES | -- | YES (n=unreported) [R4] | YES [R4] |
| Mekonen [30] (2024) | NO | 7-day Physical Activity Recall (PAR) [R5] | YES (n=160) [R6] | YES [R6] Spearman CC 0.75 (95%CI: 0.67–0.81) |
| Molvær [33] (2014) | -- | -- | -- | -- |
| Negash [37] (2022) | YES | -- | YES, unreported | -- |
| Tola [26] (2021) | N/A | N/A | N/A | N/A |
| Waaijman [41] (2014) | N/A | N/A | N/A | N/A |
| Woldemariam [32] (2020) | YES | -- | YES (n=8 D) | -- |
| Yazdanpanah [27] (2018) | YES | -- | -- | -- |
| Yazdanpanah [31] (2024) | YES | -- | -- | -- |

**Supp. Table 3 References**[R1] Macek P, Terek-Derszniak M, Zak M, Biskup M, Ciepiela P, Krol H, Smok-Kalwat J, Gozdz S, WHO recommendations on physical activity versus compliance rate within a specific urban population as assessed through IPAQ survey: a cross-sectional cohort study. BMJ Open. 2019;9(6):e028334. <https://doi.org/10.1136/bmjopen-2018-028334>
[R2] Riley L, Guhold R, Cowan M, Savin S, Bhatti L, The World Health Organization STEPwise approach to noncommunicable disease risk-factor surveillance: Methods, challenges, and opportunities. Am. J. Pub. Health 2016; 106:74–78. <https://doi.org/10.2105/AJPH.2015.302962>
[R3] Andersen LG, Groenvold M, Jørgensen T, Aadahl M. Construct validity of a revised Physical Activity Scale and testing by cognitive interviewing. Scand J Public Health 2010; 38:707–14. <https://doi.org/10.1177/1403494810380099>
[R4] Del Aguila MA, Assessment of physical activity in patients with diabetes (Lower extremities, dpaq, test reliablity). Dissertation Abstr. Int. 5–06, Section: B, 2708.
[R5] Sallis JF, Haskell, WL, Wood , PD, Fortmann, SP, Rogers, T, Blair, SN, and Paffenbarger, R, Physical activity assessment methodology in the Five City Project. American Journal of Epidemiology 1985; 121:91–106. <https://doi.org/10.1093/oxfordjournals.aje.a113987>
[R6] Zuazagoita A, Montoya I, Grandes G, Arietaleanizbeascoa M, Arce V, Martinez V et al., Reliability and validity of the 7-day Physical Activity Recall interview in a Spanish population. Eur. J. Sports Sci. 2014; 14:S361–368. <https://doi.org/10.1080/17461391.2012.705332>

**Supp. Table 4 Study participant demographics: Diabetes and wound type**

|  | **DM type** | | **DFU location** | | **DFU thickness** | |  | **Deep tissue** |  |
| --- | --- | --- | --- | --- | --- | --- | --- | --- | --- |
| **Lead author (year)** | **T1DM** | **T2DM** | **Dorsal** | **Plantar** | **Partial** | **Full** | **Infection** | **involvement** | **Ulcer type** |
| Abdissa [34] (2020) | 0.0% | 100.0% | -- | -- | O | P | -- | -- | Foot ulcer (excl. ankle) |
| Armstrong [39] (2004) | -- | -- | -- | -- | -- | -- | -- | -- | Foot ulcer |
| Badedi [28] (2019) | 0.0% | 100.0% | -- | -- | P | P | -- | -- | New OR poorly healing OR non-healing |
| Bagheri [38] (2025) ^1^ | 6.2% | 93.8% | -- | -- | -- | -- | P | -- | Foot OR ankle ulcer |
| Hirpa [35] (2023) | 28.4% | 71.6% | -- | -- | -- | -- | -- | -- | Foot ulcer (excl. ankle) |
| Jahan [36] (2023) | 0.0% | 100.0% | -- | -- | -- | -- | -- | -- | -- |
| Orlando [29] (2021) | 14.6% | 85.4% | O | P | O | P | -- | -- | Foot ulcer (excl. ankle) |
| LeMaster [40] (2008) | -- | -- | -- | -- | -- | -- | -- | -- | -- |
| Mekonen [30] (2024) | 23.5% | 76.5% | -- | -- | P | P | -- | -- | Foot ulcer (excl. ankle) |
| Molvær [33] (2014) | -- | -- | -- | -- | -- | -- | -- | -- | Foot ulcer |
| Negash [37] (2022) | 25.8% | 74.2% | -- | -- | -- | -- | -- | -- | -- |
| Tola [26] (2021) | 0.0% | 100.0% | -- | -- | -- | -- | P | P | Lower limb |
| Waaijman [41] (2014) | 28.7% | 71.3% | O | P | O | P | P | -- | Recurrent ulcer |
| Woldemariam [32] (2020) ^2^ | 30.2% 27.8% | 69.8% 72.2% | -- | -- | -- | -- | -- | -- | -- |
| Yazdanpanah [27] (2018) | 2.5% | 97.5% | -- | -- | -- | -- | -- | -- | -- |
| Yazdanpanah [31] (2024) | -- | -- | -- | -- | -- | -- | -- | -- | -- |

KEY
^1^ Requiring a healing period of at least 14 days ^2^ Demographics provided for cases then controls.

**Supp. Table 5 Demographics of physical activity with DFU outcome studies (Age & Sex)**

|  |  | **Age (years)** | | | | | | | |
| --- | --- | --- | --- | --- | --- | --- | --- | --- | --- |
| **Lead author (year)** | **Female (%)** | **Mean  ± SD** | **<30  (%)** | **30-39 (%)** | **40-49 (%)** | **≤44  (%)** | **≥50 (%)** | **45-64 (%)** | **≥65 (%)** |
| Abdissa [34] (2020) | 40.4% | -- | 11.6 | 8.7 | 26.0 | -- | 53.8 | -- | -- |
| Armstrong [39] (2004) | 5.0% | 68.5 ± 10.0 | -- | -- | -- | -- | -- | -- | -- |
| Badedi [28] (2019) ^1^ | 40.6% | -- | -- | -- | -- | 19.8 | -- | 59.8 | 20.4 |
| Bagheri [38] (2025) | 41.0% | 62.0 ± 10.2 | -- | -- | -- | -- | -- | -- | -- |
| Hirpa [35] (2023) | 50.7% | -- | 20.9 | 44.2% | | -- | 34.9 | -- | -- |
| Jahan [36] (2023) | 50.5% | 57.3 ± 10.3 | -- | -- | -- | -- | -- | -- | -- |
| Orlando [29] (2021) ^2^ | 41.7% | 72.6 ± 9.5 | -- | -- | -- | -- | -- | -- | -- |
| LeMaster [40] (2008) ^3^ | 23.0% | 62.5 ± 9.0 | -- | -- | -- | -- | -- | -- | -- |
| Mekonen [30] (2024) ^4^ | 46.4% | -- | -- | -- | -- | -- | -- | -- | -- |
| Molvær [33] (2014) | 47.6% | 64.5 ± 12.3 | -- | -- | -- | -- | -- | -- | -- |
| Negash [37] (2022) | 44.6% | 49.9 ± 15.7 | 14.2 | 16.5 | 18.0 | -- | 51.3 | -- | -- |
| Tola [26] (2021) ^5^ | 42.8% | -- | 9.8 | 31.1 | | | -- | 59.2 | |
| Waaijman [41] (2014) | 17.5% | 63.3 ± 10.1 | -- | -- | -- | -- | -- | -- | -- |
| Woldemariam [32] (2020) | 47.2% (DFU) 49.1% (Without) | 50.6 ± 6.3 (DFU) 51.5 ± 16.6 (Without) | -- | -- | -- | -- | -- | -- | -- |
| Yazdanpanah [27] (2018) | 58.1% | 53.6 ± 10.8 | -- | -- | -- | -- | -- | -- | -- |
| Yazdanpanah [31] (2024) | 58.5% | 53.2 ± 11.5 | -- | -- | -- | -- | -- | -- | -- |

**KEY**^1^ Sex calculated from Table 1 by adding together female cases and controls (42+89=131) divided by total study population (66+126+42+89=323)
Ages calculated by adding together cases and controls:≤44 yrs (21+43=64); 45-64yrs (55+138=193); ≥65yrs (32+34=66) divided by total study population
^2^ Percentages calculated from 102 males and 73 females (102+73=175 total, 73/175x100=41.7%).
^3^ Percentage of female participants provided in LeMaster’s Table 1  ^4^ Study used a different age range classification: 18-27: 25.6%; 38-47: 4.7%; 48-67: 34.9%; >68: 34.9%
^5^ Age calculated from Table 4 by adding together cases and controls: 15-29yrs (6+43=49); 30-44yrs: (23+133=156); 45-59 yrs: (29+122=151); >60 yrs: (48+98=146) divided by total study population (502).

**Supp. Table 6 Demographics of physical activity with DFU outcome studies (BMI)**

|  | **Body Mass Index (BMI) (kg/m^2^)** | | | | |
| --- | --- | --- | --- | --- | --- |
| **Lead author (year)** | **Mean ± SD** | **Underweight <18.5** | **Normal 18.5-24.9** | **Overweight 25.0-29.9** | **Obese ≥30.0** |
| Abdissa [34] (2020) | -- | 10.5% | 68.2% | 15.9% | 5.4% |
| Armstrong [39] (2004) | 30.0 ± 3.0 | -- | -- | -- | -- |
| Badedi [28] (2019) ^1^ | -- | 1.2% | 18.3% | 42.1% | 38.1% |
| Bagheri [38] (2025) | 27.9 ± 4.7 | -- | -- | -- | -- |
| Hirpa [35] (2023) | -- | -- | 58.3% | 41.7% | |
| Jahan [36] (2023) | -- | -- | -- | -- | -- |
| Orlando [29] (2021) | 30.2 ± 6.0 | -- | -- | -- | -- |
| LeMaster [40] (2008) | -- | -- | -- | -- | -- |
| Mekonen [30] (2024) | -- | -- | 82.8% | 14.2% | 3.0% |
| Molvær [33] (2014) | 30.1 ± 5.1 | -- | -- | -- | -- |
| Negash [37] (2022) | -- | 7.9% | 41.2% | 35.2% | 15.7% |
| Tola [26] (2021) | -- | -- | -- | -- | -- |
| Waaijman [41] (2014) | 30.7 ± 5.7 | -- | -- | -- | -- |
| Woldemariam [32] (2020) | -- | 9.3% | 26.1% | 37.9% | 26.7% |
| Yazdanpanah [27] (2018) | 28.7 ± 4.4 | -- | -- | -- | -- |
| Yazdanpanah [31] (2024) | 28.6 ± 4.7 | -- | -- | -- | -- |

**KEY**^1^ BMI percentages calculated by adding together case and control numbers: <18.5: (1+3=4); 18.5-24.9: (22+37=59); 25.0-29.9: (39+97=136); ≥30.0: (45+78=123) as a percentage of total study population (323). We note the total of figures supplied added up to 322.
Woldemariam: Percentages calculated from Supp. Table 2 combined case and control numbers as percentage of total study population.

**Supp. Table 7 Demographics of physical activity with DFU outcome studies (Duration of diabetes)**

|  | **Duration of diabetes (years)** | | | |
| --- | --- | --- | --- | --- |
| **Lead author (year)** | **Mean ± SD** | **< 5** | **5-10** | **≥10** |
| Abdissa [34] (2020) | -- | 56.3% | 26.4% | 17.3% |
| Armstrong [39] (2004) | 13.7 ± 9.3 | -- | -- | -- |
| Badedi [28] (2019) ^1^ | -- | 6.2% | 31.3% | 62.5% |
| Bagheri [38] (2025) | 15.8 ± 10.1 | -- | -- | -- |
| Hirpa [35] (2023) | -- | 58.3% | 25.9% | 15.8% |
| Jahan [36] (2023) | -- | -- | -- | -- |
| Orlando [29] (2021) | 21.6 ± 9.1 | -- | -- | -- |
| LeMaster [40] (2008) | -- | -- | -- | -- |
| Mekonen [30] (2024) | -- | 87.0% | | 13.0% |
| Molvær [33] (2014) | 7.0● | -- | -- | -- |
| Negash [37] (2022) | -- | 50.9% | 19.9% | 29.2% |
| Tola [26] (2021) | 28.0● (14-40) | -- | -- | -- |
| Waaijman [41] (2014) | 17.3 ± 13.5 | -- | -- | -- |
| Woldemariam [32] (2020) ^2^ | 13.0 ± 8.4 12.5 ± 9.5 | -- | -- | -- |
| Yazdanpanah [27] (2018) | 8.9 ± 6.9 | -- | -- | -- |
| Yazdanpanah [31] (2024) | 9.4 ± 6.8 |  |  |  |

**KEY**● Median rather than mean with interquartile range in brackets thereafter if available
^1^ Duration of diabetes percentages calculated by adding together case and control numbers:<5 yrs (2+18=20); 5-10yrs: (11+90=101); ≥10 yrs: (95+107=202) as a percentage of total study population (323).
^2^ Mean duration of diabetes with standard deviation (SD) shown for cases then controls.

**Supp. Table 8 Demographics of physical activity with DFU outcome studies (Ethnicity)**

|  | **Ethnicity of study cohort reporting** | |
| --- | --- | --- |
| **Lead author (year)** | **Reported?** | **Ethnicity breakdown** |
| Abdissa [34] (2020) | NO | -- |
| Armstrong [39] (2004) | NO | -- |
| Badedi [28] (2019) | NO | -- |
| Bagheri [38] (2025) | NO | -- |
| Hirpa [35] (2023) | YES | 81.7% Oromo; 15.5% Amhara; Others 2.9% |
| Jahan [36] (2023) | NO | -- |
| Orlando [29] (2021) | YES | 100% Caucasian |
| LeMaster [40] (2008) | NO | -- |
| Mekonen [30] (2024) | NO | -- |
| Molvær [33] (2014) | NO | -- |
| Negash [37] (2022) | NO | -- |
| Tola [26] (2021) | NO | -- |
| Waaijman [41] (2014) | NO | -- |
| Woldemariam [32] (2020) | NO | -- |
| Yazdanpanah [27] (2018) | YES | 50.7% Arab; 16.6% Lor; 29.3% Fars; 3.4% Other |
| Yazdanpanah [31] (2024) | YES | 55.5% Arab; 15.2% Lor; 26.7% Fars; 2.6% Other |

**KEY**
-- = unreported.

**Supp. Table 9 Loss of protective sensation testing in studies of physical activity with DFU outcomes**

| **Lead author (year)** | **Peripheral neuropathy definition** |
| --- | --- |
| Abdissa [34] (2020) | MNSI questionnaire (history) score ≥7 AND  MNSI questionnaire (exam) score ≥ 2.5 OR leg exam abnormal responses |
| Armstrong [39] (2004) | VPT > 25V |
| Badedi [28] (2019) | 10g monofilament, vibration (tuning fork) and temperature sensation (details unreported) |
| Bagheri [38] (2025) | 10g monofilament (details unreported) |
| Hirpa [35] (2023) | MNSI questionnaire (exam) score ≥ 2.5 |
| Jahan [36] (2023) | -- |
| Orlando [29] (2021) | Electromyography of peroneal motor nerve and sural sensory nerve |
| LeMaster [40] (2008) | 10g monofilament absent at any point on either foot |
| Mekonen [30] (2024) | MNSI questionnaire (exam) score ≥ 2.5 |
| Molvær [33] (2014) | -- |
| Negash [37] (2022) | -- |
| Tola [26] (2021) | -- |
| Waaijman [41] (2014) | 10g monofilament AND VPT (details unreported) |
| Woldemariam [32] (2020) | 10g monofilament (details unreported) |
| Yazdanpanah [27] (2018) | VPT (details unreported) |
| Yazdanpanah [31] (2024) | -- |

**KEY**
-- = Unreported. g = grams; MNSI = Michigan Neuropathy Screening Instrument; V= Volts; VPT = Vibration Perception Threshold

**Supp. Table 10 Foot deformity in studies of physical activity with DFU outcomes**

|  | **% cohort with deformity** | **Hammer toes** | **Claw  toes** | **Prominent MTHs** | **Amputations** | **Hallux valgus** | **Pes cavus** | **Pes planus** | **Pes equinus** |
| --- | --- | --- | --- | --- | --- | --- | --- | --- | --- |
| **Lead author (year)** |  |  |  |  |  |  |  |  |  |
| Abdissa [34] (2020) | 35.0% | 🗸 | 🗸 | 🗸 | 🗸 | 🗸 | -- | -- | -- |
| Armstrong [39] (2004) | -- | -- | -- | -- | -- | -- | -- | -- | -- |
| Badedi [28] (2019) | -- | -- | -- | -- | -- | -- | -- | -- | -- |
| Bagheri [38] (2025) | -- | -- | -- | -- | -- | -- | -- | -- | -- |
| Hirpa [35] (2023) | 74.8% | 🗸 | 🗸^1^ | 🗸 | 🗸 | 🗸 | -- | -- | -- |
| Jahan [36] (2023) | -- | -- | -- | -- | 🗸 | -- | -- | -- | -- |
| Orlando [29] (2021) | 33.1% | 🗸 | 🗸 | 🗸 | 🗸 | -- | 🗸 | -- | -- |
| LeMaster [40] (2008) | -- | -- | -- | -- | -- | -- | -- | -- | -- |
| Mekonen [30] (2024) | -- | -- | -- | -- | -- | -- | -- | -- | -- |
| Molvær [33] (2014) | -- | -- | -- | -- | -- | -- | -- | -- | -- |
| Negash [37] (2022) | -- | -- | -- | -- | -- | -- | -- | -- | -- |
| Tola [26] (2021) | -- | -- | -- | -- | -- | -- | -- | -- | -- |
| Waaijman [41] (2014) ^2^ | 90.1% | 🗸 | 🗸 | 🗸 | 🗸 | 🗸 | 🗸 | 🗸 | 🗸 |
| Woldemariam [32] (2020) | 8.1% | -- | 🗸 | -- | -- | 🗸 | -- | -- | -- |
| Yazdanpanah [27] (2018) | 9.2% | -- | -- | -- | -- | -- | -- | -- | -- |
| Yazdanpanah [31] (2024) | 11.5% | -- | -- | -- | -- | -- | -- | -- | -- |

**KEY**
-- = Unreported. Prominent MTHs = prominent metatarsal heads; High med. Arch = High medial arch;
^1^ “nail toe” presumed to be claw toes.
^2^ Derived from combined total of mild deformity (36.8%); moderate deformity (41.5%); and severe deformity (11.7%) less absent deformity 9.9%.

**Supp. Table 11 Footwear evaluation in studies of physical activity with DFU outcomes**

|  | **% wearing therapeutic footwear** | **% wearing non-therapeutic footwear** | **Footwear fit standard** |
| --- | --- | --- | --- |
| **Lead author (year)** |  |  |  |
| Abdissa [34] (2020) | -- | -- | -- |
| Armstrong [39] (2004) | 100.0% | 0.0% | -- |
| Badedi [28] (2019)^1^ | -- | -- | -- |
| Bagheri [38] (2025) | -- | -- | -- |
| Hirpa [35] (2023) | -- | -- | -- |
| Jahan [36] (2023)^2^ | -- | -- | -- |
| Orlando [29] (2021) | 36.6% | 63.4% | -- |
| LeMaster [40] (2008)^3^ | 50.0% | 50.0% | -- |
| Mekonen [30] (2024) | -- | -- | -- |
| Molvær [33] (2014) | -- | -- | -- |
| Negash [37] (2022) ^2^ | -- | -- | -- |
| Tola [26] (2021) | -- | -- | -- |
| Waaijman [41] (2014) ^4^ | 100.0% | 0.0% | -- |
| Woldemariam [32] (2020) | -- | -- | Footwear wider than foot (tracing technique) No reddened areas of foot on shoe removal |
| Yazdanpanah [27] (2018) | -- | -- | -- |
| Yazdanpanah [31] (2024) | -- | -- | Insufficient length, width or height based on shoe size aOR 10.4 (95% CI 4.5-24.1), p<0.001 |

**KEY**
-- = Unreported; aOR = Adjusted Odds Ratio; CI = Confidence Interval.
^1^ Appropriate footwear subsumed within foot self-care including foot inspection, cleaning, drying etc.
^2^ Footwear characterised as percentage classified as ‘comfortable footwear’ which is undefined.
^3^ All 186 participants took part in a therapeutic footwear clinical trial.
^4^ Custom-made shoes or extra-depth shoes.
Total participants (22+31+42+66=161); Cases (22+31=53); Controls (42+66=108); Ill-fitting cases: (31/53*100=58.5%); Ill-fitting controls: (42/108*100=38.9%)
